# Acceptabilité du test VIH proposé aux nourrissons dans les services pédiatriques, en Côte d'Ivoire, *Significations pour la couverture du diagnostic pédiatrique* [Fn FN0001]

**DOI:** 10.1080/17290376.2014.938101

**Published:** 2014-08-04

**Authors:** Maxime Oga, Hermann Brou, Hortense Dago-Akribi, Patrick Coffie, Clarisse Amani-Bossé, Didier Ékouévi, Vincent Yapo, Hervé Menan, Camille Ndondoki, M. Timité-Konan, Valériane Leroy

**Affiliations:** ^a^ Sociologist at the Pacci Program Site ANRS, Abidjan, Côte d'Ivoire; ^b^ PhD student in the Department of Anthropology and Sociology (DAS), Alassane Ouattara University of Bouaké; ^c^ PhD, Statistician-Demographer, Project Manager at the Pacci Program Site ANRS, Abidjan, Côte d'Ivoire; ^d^ PhD, Psychologist, Teacher-researcher at the Felix Houphouet Boigny University, Abidjan-Cocody; ^e^ PhD, Epidemiologist, Researcher, Project Manager at the Pacci Program Site ANRS, Abidjan, Côte d'Ivoire; ^f^ MD, Project Manager at the Pacci Program Site ANRS, Abidjan, Côte d'Ivoire; ^g^ PhD, Epidemiologist, Scientific Director of the Pacci Program Site ANRS, Abidjan, Côte d'Ivoire; ^h^ Teacher-researcher at the Department of Public Health, Faculty of Health Sciences, University of Lomé, Togo; ^i^ Teacher-researcher at the INSERM Unit 897, Institute of Public Health, Epidemiology and Development (ISPED), University of Bordeaux 2, Bordeaux, France; ^j^ Doctor of Pharmacy, Biologist at the Laboratory of Virology, CeDReS, Abidjan, Côte d'Ivoire; ^k^ PU, Director of the Laboratory of Virology, CeDReS, Abidjan, Côte d'Ivoire; ^l^ PhD, Epidemiologist at the INSERM Unit 897, Institute of Public Health, Epidemiology and Development (ISPED), University of Bordeaux 2, Bordeaux, France; ^m^ PU, Chief of Pediatrics, Centre Hospitalier Universitaire (CHU) Yopougon, Abidjan, Côte d'Ivoire; ^n^ PU, South investigative project Pedi-Test ANRS 12165; ^o^ PhD, Epidemiologist, Teacher-researcher at the INSERM Unit 897, Institute of Public Health, Epidemiology and Development (ISPED), University of Bordeaux 2, Bordeaux, France; ^p^ PhD, Epidemiologist at the North investigative project Pedi-Test ANRS 12165

**Keywords:** Acceptabilité, Test VIH, Enfants, Nourrissons, acceptability, HIV testing, children, infants

## Abstract

*Problème:* Le dépistage VIH chez les enfants a rarement été au centre des préoccupations des chercheurs. Quand le dépistage pédiatrique a retenu l'attention, cela a été pour éclairer seulement sur les performances diagnostiques en ignorant même que le test pédiatrique comme bien d'autres peut s'accepter ou se refuser. Cet article met au cœur de son analyse les raisons qui peuvent expliquer qu'on accepte ou qu'on refuse de faire dépister son enfant.

*Objectif:* Etudier chez les parents, les mères, les facteurs explicatifs de l'acceptabilité du test VIH des nourrissons de moins de six mois.

*Méthodes:* Entretien semi-directif à passages répétés avec les parents de nourrissons de moins de six mois dans les formations sanitaires pour la pesée/vaccination et les consultations pédiatriques avec proposition systématique d'un test VIH pour leur nourrisson.

*Résultats:* Nous retenons que la réalisation effective du test pédiatrique du VIH chez le nourrisson repose sur trois éléments.

Primo, le personnel de santé par son discours (qui dénote de ses connaissances et perceptions même sur l'infection) orienté vers les mères influence leur acceptation ou non du test. Secundo, la mère qui par ses connaissances et perceptions même sur le VIH, dont le statut particulier, l'impression de bien-être chez elle et son enfant influence toute réalisation du test pédiatrique VIH. Tertio, l'environnement conjugal de la mère, particulièrement caractérisé par les rapports au sein du couple, sur la facilité de parler du test VIH et sa réalisation chez les deux parents ou chez la mère seulement sont autant de facteurs qui influencent la réalisation effective du dépistage du VIH chez l'enfant.

Le principe préventif du VIH, et le désir de faire tester l'enfant ne suffisent pas à eux seuls pour aboutir à sa réalisation effective, selon certaines mères confrontées au refus du conjoint. A l'opposé, les autres mères refusant la réalisation du test pédiatrique disent s'y opposer ; bien entendu, même dans le cas où le conjoint l'accepterait.

*Discussion:* Les mères sont les principales mises en cause et craignent les réprimandes et la stigmatisation. Le père, le conjoint peut être un obstacle, quand il s'oppose au test VIH du nourrisson, ou devenir le facilitateur de sa réalisation s'il est convaincu. Le positionnement du père demeure donc essentiel dans la question de l'acceptabilité du VIH pédiatrique. Les mères en ont conscience et présagent des difficultés à faire dépister ou non les enfants sans avis préalable du conjoint à la fois père, et chef de famille.

*Conclusion:* La question du dépistage pédiatrique du VIH, au terme de notre analyse, met en face trois éléments qui exigent une gestion globale pour assurer une couverture effective. Ces trois éléments n'existeraient pas sans s'influencer, donc ils sont constamment en interaction et empêchent ou favorisent la réalisation ou non du test pédiatrique. Aussi, dans une intention d'aboutir à une couverture effective du dépistage VIH des nourrissons, faut-il tenir compte d'une gestion harmonieuse de ces trois éléments: La première, la mère seule (avec ses connaissances, ses perceptions), son environnement conjugal (de proposition du test intégrant 1- l’époux et / ou père de l'enfant avec ses perceptions et connaissances sur l'infection 2- la facilité de parler du test et sa réalisation chez les deux ou un des parents, la mère) et les connaissances, attitudes et pratiques du personnel de l’établissement sanitaire sur l'infection du VIH.

*Recommandations:* Nos recommandations proposent une redéfinition de l'approche du VIH/sida vers des familles exposées au VIH et une intégration plus accentuée du père facilitant leur propre acceptation du test VIH et celle de leur enfant.

## Abstract (en)

*Problem:* HIV testing in children had rarely been a central concern for researchers. When pediatric tracking retained the attention, it was more to inform on the diagnosis tools performances rather than the fact the pediatric test can be accepted or refused. This article highlights the parent's reasons which explain why pediatric HIV test is accepted or refused.

*Objective:* To study among parents, the explanatory factors of the acceptability of pediatric HIV testing among infant less than six months.

*Methods:* Semi-structured interview with repeated passages in the parents of infants less than six months attending in health care facilities for the pediatric weighing/vaccination and consultations.

*Results:* We highlight that the parent's acceptance of the pediatric HIV screening is based on three elements.

Firstly, the health care workers by his speech (which indicates its own knowledge and perceptions on the infection) directed towards mothers' influences their acceptance or not of the HIV test. Secondly, the mother who by her knowledge and perceptions on HIV, whose particular status, give an impression of her own wellbeing for her and her child influences any acceptance of the pediatric HIV test. Thirdly, the marital environment of the mother, particularly characterized by the ease of communication within the couple, to speak about the HIV test and its realization for the parents or the mother only are many factors which influence the effective realization of the pediatric HIV testing.

The preventive principle of HIV transmission and the desire to realize the test in the newborn are not enough alone to lead to its effective realization, according to certain mothers confronted with the father's refusal. On the other hand, the other mothers refusing the realization of the pediatric test told to be opposed to it; of course, even if their partner would accept it.

*Discussion:* The mothers are the principal facing the pediatric HIV question and fear the reprimands and stigma. The father, the partner could be an obstacle, when he is opposed to the infant HIV testing, or also the facilitator with his realization if he is convinced. The father position thus remains essential face to the question of pediatric HIV testing acceptability. The mothers are aware of this and predict the difficulties of achieving their infant to be tested without the preliminary opinion of their partner at the same time father, and head of the family.

*Conclusion:* The issue of pediatric HIV testing, at the end of our analysis, highlights three elements which require a comprehensive management to improve the coverage of pediatric HIV test. These three elements would not exist without being influenced; therefore they are constantly in interaction and prevent or support the realization or not pediatric test. Also, with the aim to improve the pediatric HIV test coverage, it is necessary to take into account the harmonious management of these elements. Firstly, the mother alone (with her knowledge, and perceptions), its marital environment (with the proposal of the HIV test integrating (1) the partner and/or father with his perceptions and knowledge on HIV infection and (2) facility of speaking about the test and its realization at both or one about the parents, the mother) and of the knowledge, attitudes and practices about the infection of health care workers of the sanitary institution.

*Recommendations:* Our recommendations proposed taking into account a redefinition of the HIV/AIDS approach towards the families exposed to HIV and a more accentuated integration of the father facilitating their own HIV test acceptation and that of his child.

## 1. Introduction

La prévalence de l'infection à VIH chez les nouveau-nés demeure croissante. En Afrique subsaharienne, elle l'est en raison de l’épidémie VIH estimée chez les femmes à 59% (selon l'organisation onusienne : ONUSIDA). L'ONUSIDA dénombrait dans le monde environ 2,1 millions d'enfants infectés dont environ 1 407 000 enfants^1^ en Afrique Subsaharienne et 50 000 enfants infectés en Côte d'Ivoire.

Les études portant sur le dépistage du VIH se sont essentiellement intéressées, en Côte d'Ivoire comme partout en Afrique, aux adultes. Et, dans le cadre de la prévention de la transmission du VIH de la mère à l'enfant (PTME), ces études^2^ se sont plus intéressées au dépistage précoce du VIH chez les femmes enceintes en Côte d'Ivoire. Ceci étant, en 2008, en Côte d'Ivoire, seulement 34% des grossesses enregistrées ont été testées pour le VIH. D'où, la probabilité de n'avoir pas pu éviter 4 852 infections^3^ pédiatriques sur 400 981 femmes qui n'ont pas eu accès aux services de PTME. En outre, seulement 6,7% des enfants infectés par le VIH sont médicalement suivis. 4,5% prennent un traitement antirétroviral depuis 2009.^4^

Par conséquent, peu d'enfants (moins de 10% des infectés) ont accès à un traitement antirétroviral. Cela constitue un handicap à la recommandation de l'OMS.^5^ Or sans traitement, 35% des enfants infectés décèdent avant leur premier anniversaire et, 50% dans la deuxième année de vie (Newell *et al*. 2004). Il est donc urgent de sauver ces enfants, y compris ceux, exposés au VIH, qui n'ont pas encore été dépistés afin de les mettre sous traitement antirétroviral en cas d'infection confirmée.

Ainsi, tous les enfants exposés au VIH (nés de mère infectée) devraient recevoir une proposition de test. Pour cela, il est important de circonscrire chez les parents la problématique de l'acceptabilité de ce test chez leurs enfants. Saisir les significations profondes en précisant les contraintes, les difficultés de l'acceptabilité du dépistage VIH chez les nourrissons est important pour deux raisons essentielles. Primo, les raisons du diagnostic ou non chez l'enfant demeurent méconnues. Secundo, l'acceptabilité du test chez l'enfant dépend de l'avis des parents qui posent des difficultés quant à leur propre dépistage (Dédy & Tapé 1995; Oga 2006). De ce fait, il apparaît nécessaire d'appréhender auprès des parents les raisons de réaliser le test VIH ou non chez les nouveau-nés.

La question du dépistage chez les enfants a rarement ou peu été objet d’étude singulière. Et, les études relevant cette question éclairent que sur la possibilité de réaliser ou non le diagnostic quel que soit le contexte. Dans les sites où la prévalence de l’épidémie est élevée, ces études montrent que l'acceptation du test pédiatrique avoisine 90% dans les services vaccinaux (Kankasa *et al.* 2009) ou dans les services d'hospitalisations pédiatriques (Rollins *et al.* 2009).

Ces différents travaux ont, sans aucun doute, porté sur la faisabilité pratique et sur l'acceptabilité du dépistage VIH pédiatrique mais sans prendre en compte les facteurs explicatifs de cette acceptabilité. Or, le dépistage du VIH est accepté ou refusé selon des facteurs, des raisons propres aux populations. Quels justificatifs à l'acceptation du test pédiatrique par certains parents, et quelles en sont les significations profondes ? Quelles sont les significations permettant de comprendre le refus du dépistage pédiatrique par les autres ? L'objectif, ici, est de saisir, chez les parents, les raisons de l'acceptabilité du dépistage précoce des nourrissons âgés de 6 à 26 semaines.

## 2. Méthodes

Les données de cette étude ont été recueillies dans le cadre du projet Pédi-Test ANRS 12165^6^ dont l'approche, la méthode et les principaux résultats ont été décrits ailleurs (Leroy *et al*. 2009). Brièvement,

- le schéma d’étude est transversal et a permis de proposer systématiquement, aux parents, un test de dépistage en routine pour leurs enfants dans les services de consultations pédiatriques, de pesée et de vaccination dans trois structures sanitaires à Abidjan (2) et à Bonoua (1), en Côte d'Ivoire.
- La population se composait
  ○ de tous les parents, c'est-à-dire, père, mère ou tuteurs légaux de tout enfant âgé de 6 à 26 semaines, venant pour une consultation postnatale de type vaccinale, pesée ou consultation de pédiatrie générale dans l'un des centres de l’étude: la FSU-Com HKB d'Abobo Avocatier, l'hôpital général de Koumassi et l'hôpital général de Bonoua.
  ○ Echantillon d’étude, faute de n'avoir pas eu en entretien les pères et tuteurs légaux, l’échantillon de l’étude est uniquement composé des mères de ces enfants.
  ○ Sous-échantillon représentatif des strates: statut sérologique VIH, statut matrimonial et site d'enquête (*N* = 40).
- Outils de collecte des données:
  ○ Mode opératoire
    • des séances de counselling (entretien individuel) par des conseillères ont été réalisées avec les parents et portaient sur l'information et la proposition du principe de l’étude PEDI-TEST et du principe du dépistage précoce de l'enfant. Et, tous ceux qui acceptaient participer à l’étude ont été conduits chez
    • le sociologue de l’équipe pour entretien.
  ○ Pour le recueil des données, nous avons eu recours au guide d'entretien qui nous a permis de réaliser avec les parents (mères), des enfants de 6 à 26 semaines et un seul couple parent^7^ donc un seul père, des entretiens semi-directifs à passages répétés.
- Données analysées:
  ○ recueillies essentiellement par entretien semi-directif (outil qualitatif), l'analyse portant sur les données recueillies auprès des mères a aussi été qualitative.
  ○ Nous avons procédé par thème (Blanchet et Gotmann 1992). Il s'agissait de rechercher « une cohérence thématique inter-entretiens » (Blanchet et Gotmann 1992). Et, l'analyse des entretiens a été aussi illustrative.

Trois préoccupations ont retenu l'attention: (1) les interprétations que se font les mères face à la proposition du dépistage pédiatrique de leur enfant, (2) les contraintes et difficultés du test liées à l'acceptation et/ou au refus du test VIH de l'enfant (3) les nouvelles questions sur la santé infantile suscitées par ce dépistage pédiatrique.

## 3. Resultats

### 3.1. De la proposition au diagnostic pediatrique VIH

**Fig. 1.**
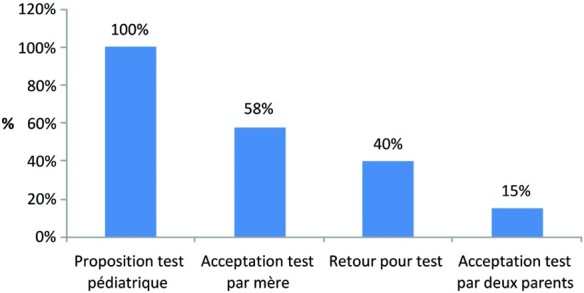
Données en cascade de la proposition à l'acceptabilité du diagnostique Pédiatrique. *Source*: Réalisé à partir des données de notre enquête sur la proposition du test pédiatrique VIH aux enfants de 6 à 26 mois.

Dans l'ensemble, il est à constater une modification considérable des proportions de la proposition (100%) à l'acceptation du test VIH pédiatrique par les parents en passant par l'acceptation maternelle (58%), le retour pour le test de l'enfant de 6 à 26 semaines (40%) et l'acceptation du test par les deux parents (Fig. 1).

### 3.2. Plus de mères que de couples parents en entretien

35 entretiens réalisés et répartis comme suit: 1 couple parent séroconcordant VIH-négatif, 15 mères VIH-négatif et 18 mères de statut VIH-inconnu. De ces enquêtés, 20 mères et le couple parents ont accepté le test pédiatrique. Des 20 mères acceptant le test de l'enfant, on comptait 7 de statut VIH-négatif et 13 mères de statut VIH-inconnu. 13 mères (8 séronégatives et 5 de statut VIH-inconnu) ont refusé de dépister leur nouveau-né. L’âge des enquêtés variait entre 18 et 38 ans avec un niveau d’étude allant du primaire (19 mères) au secondaire (premier cycle, 9 mères), (second cycle, 7 enquêtés). Des 35 enquêtés, l'on a 5 mariages légaux, 10 mariages coutumiers, 15 unions libres, 5 célibataires.

Dans l'ensemble, 33 mères et un couple dont 1 père ont eu des entretiens. Les résultats dont nous faisons mention dans cet article-ci se sont basés uniquement sur les propos des mères et, quel que soit leur choix, elles invoquent leurs raisons. Celles-ci peuvent se ressembler ou s'exclure.

### 3.3. Les motivations communes aux decisions

Elles se fondent essentiellement sur deux faits, la bonne santé de l'enfant (impression de bien être) et le statut particulier accordé à l'infection, sans obéir aux mêmes logiques.

#### 3.3.1. L'impression de bien être ou bonne santé de l'enfant

Accepter le dépistage pédiatrique pour « la bonne santé de l'enfant » révèle deux logiques: (1) la volonté manifeste des parents d'adhérer au principe préventif de l'infection, (2) l'acceptation du test chez l'enfant dont les parents se convainquent, de toutes façons, qu'il sera dépisté non-infecté:

« [ … ] affaire de test de sida là, façon les femmes nous ont parlé, je pense que c'est bon de faire [ … ] parce si tu ne fais pas tu ne peux pas savoir la vérité et puis il faut faire parce qu'on ne sait jamais [ … ] / [ … ] D'ailleurs même, moi, j'ai déjà fait le test une fois. Aujourd'hui encore j'ai fait et puis j'ai accepté pour lui parce que l'enfant ne peut pas avoir sida. Toi-même regarde (exhibition de l'enfant, présentation à l'enquêteur) est-ce que on peut dire que enfant comme çà là a sida ? », KA-378, VIH-négatif.

Ces parents jouent la prudence, pour eux, l'enfant en apparente bonne santé ne peut être soupçonné d'infection. Néanmoins, ils acceptent le test pour vérifier et conforter leur sentiment.

Par contre, les parents refusant le test de l'enfant évoquent sa ‘bonne santé’ pour signifier le lien entre leur propre santé et celui de l'enfant. Du coup, l'apparente bonne santé dont ils font preuve ne leur permet pas d'accepter de soumettre l'enfant au test.

« … pourquoi faire son test [ … ] nous (père et mère) aussi, nous sommes en bonne santé et on demande de faire test de sida de l'enfant. Ça je ne peux pas accepter. » BR-256, VIH-inconnu

Pour ces mères, l'embonpoint de l'enfant est non seulement un indicateur de bonne santé mais aide à la décision. Lorsque l'enfant présente une apparence reluisante, elles ne voient aucun intérêt à lui faire le test puisqu'aucun doute sur sa probable infection ne les anime.

#### 3.3.2. Stigmatisation du statut particulier du VIH et résignation

L'infection à VIH fait peur à tous ainsi qu'aux parents à qui le dépistage pédiatrique est proposé. Son statut particulier (à la différence des autres pathologies) accroit davantage la peur, les craintes, et conduit les parents à refuser toute intervention liée au VIH car influe négativement sur la perception des mères. Par ailleurs, et comme celles qui acceptent le test, les mères qui le refusent s'en remettent au personnel médical:

« Quand mes enfants sont malades, le médecin fait pour eux ce qu'il faut sans demander … même quand il demande, c'est pour savoir si on peut payer les médicaments … mais pour le sida on pose toujours des questions pour nous effrayer … nous on ne sait rien c'est pour cela qu'on vient à l'hôpital … » Florence A. 29 ans, VIH-inconnu, mariage coutumier.

Pour certaines mères, le statut particulier du VIH justifie l'acceptabilité du test pédiatrique. En effet, les mères qui l'acceptent, tout comme celles qui le refusent, s'interrogent sur la nécessité d'accorder à l'infection un statut particulier:

« Pourquoi pour les autres maladies on ne demande pas si je veux ou bien je ne veux pas … et puis pour sida là on me parle longtemps, on demande si je peux ou bien je ne peux pas payer, pourquoi on ne fait pas la même chose et puis c'est sur sida là seulement on fait çà. Tout çà selon moi fait peur et quand c'est comme cela [ … ] les gens refusent, ils ont raison » Colombe 32 ans, mariage coutumier, VIH-négatif.

Les propos de cette mère révèlent que les mères qui ont accepté le dépistage pédiatrique ne partagent pas cette particularité accordée au VIH. Pour elles, de même qu'aucun avis favorable n'est requis par le personnel de santé lors des consultations d'autres maladies, de même aussi aucun avis favorable ne devait être requis par lui dans le cas du VIH avant toute intervention. Selon elles, cette procédure qui subordonne le test pédiatrique à l'autorisation préalable des parents, expliquent en partie la réticence/refus. De l'avis des enquêtées, il revient aux professionnels de donner des explications aux enquêtées sur les soupçons établis suites au diagnostic et de tout mettre en œuvre pour l'amélioration de l’état de santé du patient et non de lui demander et/ou d'attendre son avis pour la réalisation du soin qu'exige le diagnostic.

### 3.4. Les motivations propres a chaque decision

#### 3.4.1. Santé de l'enfant et des parents au centre du test pédiatrique VIH

En amont de la décision du test se trouvent des motivations portant sur l'enfant, les parents. Les mères qui ont accepté le dépistage pédiatrique évoquent: la santé de l'enfant, la volonté de connaître son statut et éventuellement, la PEC en cas d'infection. Pour ces mères, il est important d'assurer à l'enfant un bien être par sa PEC s'il est infecté. Cela commence par le test.

Deux autres raisons, sont liées aux parents: (1) la psychose créée par le sida chez les mères. Ici, la réalité est que le VIH/sida régulièrement évoquée obsède les parents. Cette situation a l'avantage de pousser les parents à accepter le dépistage pédiatrique avec une double intention: (i) se libérer de leur angoisse (ii) la possibilité pour l'enfant de bénéficier d'une PEC précoce, en cas d'infection.

« … Hum … comme on dit qu'il y a médicaments pour soigner les enfants et même les grandes personnes. Et puis chaque fois on parle de test de sida. À la télévision, c'est de çà seulement qu'on parle. Quand j’étais enceinte, on m'a parlé de çà, j'ai eu peur et je n'ai pas fait. Aujourd'hui encore je viens à l'hôpital … on me parle de test, vraiment je ne peux plus, je suis fatigué d'entendre cela et ne rien faire … », Christine mariage civil 35 ans de statut VIH inconnu.

(2) La perception indirecte du statut VIH des parents via le résultat de l'enfant. Le résultat du test de l'un des parents est supposé être celui de l'enfant. C'est aussi le cas du résultat de la femme enceinte que s'approprie le conjoint comme étant sien,

« … j'ai dit qu’à l'hôpital on m'a demandé de faire le test et j'ai accepté de faire. Bon après, il n'a rien dit et j'ai montré le papier de mon résultat. Il n'a rien dit mais il était content quand il a vu le résultat et il a dit c'est que lui aussi il est négatif. Mais, je ne sais s'il est allé faire son test après. Maintenant comme j'ai fait et puis je n'ai rien c'est que mon mari n'a rien et puis l'enfant que j'ai eu avec lui aussi n'a rien. » Alexise 38 ans, mariage coutumier, VIH-négatif.

#### 3.4.2. Le trop jeune âge incite au refus du dépistage pédiatrique

Les mères refusant le test de l'enfant estiment qu'il serait *‘trop petit’*. Cette conception est due à la masse, l’âge, l'innocence et aux piqûres d'aiguilles. Dans la première situation, l'enfant est dit innocent. Par *‘innocence de l'enfant’*, les mères traduisent l'idée selon laquelle on ne peut être infecté qu'après *prise de risques*. Or le nourrisson ‘*n'a pris aucun risque*’. Une telle approche montre les limites des connaissances des enquêtées sur les modes de transmission VIH notamment ceux sur la TME. Ensuite, la question relative aux piqûres d'aiguilles fait allusion au prélèvement sanguin pour le test. Il traduit la peur des parents face aux injections en général, pour eux-mêmes et à fortiori pour leur jeune enfant. Enfin, la masse et l’âge des enfants expliquent aussi le refus du dépistage pédiatrique:

« … Je refuse de faire maintenant le dépistage de ma fille parce qu'elle n'est pas grosse, elle est trop petite, pèse pas. Or pour faire le test on prend du sang, voilà pourquoi je ne veux pas faire maintenant le dépistage de ma fille. Ma fille même a quoi à voir dans affaire de sida là [ … ]? Elle est trop petite pour ça. Quelqu'un comme ça qui ne sait rien de tout ce qui se passe là, c'est à elle on va demander de faire test de sida. Vraiment pour moi ça ne doit pas être possible. Et aussi j'ai peur de dépister mon enfant alors que son père n'est pas informé, n'a pas donné son accord … » Anastasie 30 ans, concubine, VIH-inconnu.

#### 3.4.3. L'inexistence de remède et la spontanéité de la proposition

Le dépistage chez l'enfant est refusé par les mères pour deux autres raisons, (i) se présenté à l'hôpital pour une raison autre que le test de l'enfant explique parfois son report ou son refus:

« … Bon affaire de dépistage là, vraiment, moi je ne savais pas cela avant. Je viens à l'hôpital pour vite faire la pesée de l'enfant, aller au marché et partir à la maison pour mes activités ménagères. C'est maintenant qu'on vient me parler de test de l'enfant. Vraiment ce sera la prochaine fois, aujourd'hui, je n'ai pas pensé à cela … ».

(ii) l'inexistence de remède, l'inopportunité de connaitre le statut VIH de l'enfant:

« Je sais que faire test à l'enfant est une bonne chose. Mais mon problème, c'est qu'après si l'enfant est infecté on ne va pas le guérir. Tout ce qu'on peut faire, c'est de faire en sorte qu'il vive encore un-peu et puis il va mourir. Donc si c'est pour voir l'enfant souffrir et puis mourir après, pourquoi chercher à savoir ce qu'il a ? Moi, je ne souhaite rien savoir sur mon statut et celui de l'enfant tant que l'on n'a pas encore de médicament pour guérir. Pour moi, cela ne sert à rien de savoir que son enfant est infecté et attendre qu'il meure à petit feu. »

Ces différentes illustrations montrent que le refus des mères de dépister l'enfant est non seulement dû à la peur de savoir leur enfant infecté mais aussi à l'inexistence de remède. D'où, l'expression de leur impuissance dans l’éventualité d'un résultat positif.

Par ailleurs, en plus de ces raisons mentionnées plus haut, quels autres déterminants à l'acceptabilité du test pédiatrique ? Quelles peuvent-être les contraintes des mères face au test pédiatrique ?

### 3.5. Environnement conjugal, entre contraintes et difficultés

#### 3.5.1. Il n'est pas toujours facile d'en parler … 

La quasi-totalité des enfants en consultation sont accompagnés par leurs mères seules. Il leur a été proposé de dépister l'enfant et se sont chargées d'informer le conjoint. Les mères l'ont fait à la demande du projet mais aussi par obligation pour accomplir leur devoir vis-à-vis du conjoint. Théry I a montré que la vie en couple impliquait le ‘devoir de dire’ (Raynaut & Muhongayire 1994; Théry 1999). Cependant ce ‘devoir de dire’ ne peut se faire sans anticiper sur les difficultés (ou les réactions liées à ce que l'on va dire), comme c'est le cas dans l'annonce de sa séropositivité au conjoint (Coulibaly *et al.* 2003; Desclaux 2004; Hassoum 2006; Medley *et al.* 2004; Raynaut & Muhongayire 1994; Tijou-Traoré & Groupe Ditrame Plus 3 Dialogue 2006; Vidal 2000).

Avec les couples ayant déjà entamé des échanges sur le VIH/SIDA, les mères se sentent plus à l'aise pour parler de la proposition du test pédiatrique au père. Ce qui n'est pas le cas dans les autres couples où le sida est sujet tabou. Ainsi, quand le conjoint reste attentif, les mères chez qui la difficulté est surmontée affirment pouvoir informer parce que les échanges antérieurs les rassurent:

« Non je pense que ça ne sera pas difficile à dire [ … ]. Ça sera facile de dire parce qu'on parle de ça déjà et c'est la santé du bébé aussi dê. Ça sera facile aussi parce que souvent quand je lui dis quelque chose il accepte, il ne parle pas … » Nat 26 ans, concubine, VIH-négatif.

Beaucoup plus de mères, dont les conjoints ont déjà fait leur dépistage, se sentent rassurées d'informer l’époux sur le test pédiatrique:

« Je pense que mon mari va accepter le dépistage de notre enfant. Il va accepter puisqu'il a déjà fait lui-même son test [ … ] m'a informé [ … ] et il m'a présenté son résultat aussi. » Anne, concubine, VIH-inconnu, accepte test.

Par contre, celles pour qui la difficulté est encore vivace ont deux attitudes. (1) continuer d'en parler tant que le père manifeste de l'intérêt ou de la réceptivité à aborder le sujet. Dans le cas contraire, la mère renonce:

« … Vraiment affaire de test de sida là, je ne sais pas mais je vais essayer. Si mon mari ne dit rien, je vais continuer jusqu’à ce qu'il accepte. Maintenant comme son affaire est compliquée, s'il commence à bavarder ah moi je ne vais plus parler de test de l'enfant … » Val, 35 ans, mariage coutumier, statut VIH-inconnu, accepte test.

(2) ne pas informer le conjoint, le père car tout débat autour du VIH/SIDA est toujours mal apprécié:

« … vraiment, je vais vous dire que je ne peux même pas lui parler de test ou même de ce qui se rapproche du sida. Je ne peux pas faire ça pour éviter les histoires. Maintenant si Dieu fait qu'un jour on vient ici ensemble, bon vous allez lui parler … Là on évite les histoires qui peuvent conduire au renvoie ou à bouder son camarade » Aïc, 24 ans, VIH-inconnu, accepte test.

Quelles que soient leurs expériences, craintes, situations vécues, connaissances sur la TME, les mères ayant reçu la proposition du test pédiatrique font face à la difficulté de parler de ce dépistage et renseignent sur le type de rapport entretenu dans leur couple. Cette difficulté les contraint à user de stratégies. Quelles sont donc ces stratégies employées par les mères face au test de leur enfant ? Et quels peuvent en être les risques ?

#### 3.5.2. … Certaines mères favorables au dépistage pédiatrique utilisent des stratégies

Elles sont de deux ordres, *insistance/soumission*, *insistance/dissimulation* ou *insistance/défiance*. L*’insistance/soumission* est utilisée par les mères favorables au dépistage de leur enfant. Dans une première étape, elles comptent informer leur conjoint puis insister pour obtenir son accord. Il faut insister pour voir son souhait agréer:

« … avec les différents rendez vaccinaux et des CPN je ne décide pas toute seule de l'amener à l'hôpital ou de venir pour mes CPN. La décision d'envoyer l'enfant à l'hôpital ou de venir faire mes CPN se prend par mon homme qui me donne les moyens qu'il faut pour nous rendre à l'hôpital après avoir compris qu'il faut vraiment aller à l'hôpital. Donc moi, ce que je fais c'est que j'informe mon mari et j'insiste que l'enfant a ses RDV ou bien l'enfant a ses vaccins à faire. Je lui fais comprendre que c'est bien de faire suivre l'enfant en respectant tous les RDV et la mère aussi même après accouchement. Là comme ça, les sages-femmes et les médecins les suivent pour leur bonne santé. » FA 116, 31 ans, VIH-négatif.

L'insistance, pour ces mères, vise à se faire comprendre et à faire accepter leur souhait. C'est une stratégie utilisée dans des cas similaires de maladies infantiles (Suremain, Lefèvre & Pécho 2000). Il faut insister parce que les deux conjoints n'ont pas la même perception de la question débattue/de la gravité du mal ou de la nécessité des soins chez l'enfant (Baxerres & Le Hesran 2004; Suremain *et al.* 2000; Tijou-Traoré 2009).

Cependant, elles avouent être obligées de se soumettre à l'avis de l’époux quel qu'il soit. Pour ces mères, si l'insistance permet de se faire comprendre ou de faire accepter son souhait, il n'est pas admissible de faire le test de l'enfant en passant outre la décision du père. Il faut se soumettre à sa décision parce que l’époux est à la fois père, chef de famille et la préservation de la stabilité du couple reste préoccupante. En tant que père, le conjoint est le premier responsable de l'enfant au plan légal et traditionnel. Ainsi, pour les mères, il est bien de ne pas prendre d'initiative contraire à celle de l’époux surtout pour le VIH. Par conséquent, la soumission à la décision de l’époux permet d’éviter des soucis conjugaux et d'entretenir la stabilité dans le couple.

*L'insistance/dissimulation* ou *l'insistance/défiance* sont utilisées par les mères favorables au dépistage pédiatrique. Elles procèdent par insistance et en cas de refus du conjoint, elles se cachent ou le défient pour faire le test de leur enfant avec pour raison: l'expérience antérieure des échanges sur le VIH/sida. Or pour elle, retarder le dépistage peut être préjudiciable à l'enfant considéré comme seul ‘bien’ ou ‘bénéfice’:

« … L'enfant représente tout ce qui revient à la femme quand le mari va partir [ … ] Les hommes sont comme ça, ils nous marient et nous abandonnent. Donc si la femme ne s'occupe pas bien de son enfant, elle va sortir zéro … » Pierrette, VIH-négatif, mariage coutumier.

Les mères jugent, au regard des échanges déjà engagées, que le conjoint peut refuser la proposition de dépister l'enfant ou l'accepter mais tardivement. Pour elles donc, il leur faut prêter plus d'attention à l'enfant et ne ménager aucun effort pour son bien-être car le contexte ivoirien est marqué par la seule reconnaissance du mariage civil qui offre aux enfants et à leur mère des garanties après le divorce. Ce qui n'est pas le cas avec le mariage traditionnel. D'où la défiance du père pour garantir à l'enfant une bonne santé en procédant en cachette à son dépistage tout en continuant la discussion avec le père. Toutefois, elles comptent lui avouer une fois en possession du résultat:

« [ … ] Son père ne peut pas refuser le test de son enfant s'il l'aime vraiment. Et de toutes les façons, comme je sais que faire le test à notre enfant est une bonne chose, si mon mari refuse je ne vais pas m'opposer à lui comme ça. Mais je vais tout faire pour qu'on fasse le test de sida de mon enfant. Si même, je dois faire signer ce papier par quelqu'un d'autre, je vais le faire. Pour moi, il faut tout faire pour le dépistage de l'enfant. Maintenant, une fois le test est fait, je vais continuer de discuter avec le père comme si j'avais suivi sa décision. Et si après il accepte je fais semblant d'aller à l'hôpital et je reviens pour lui donner le résultat, c'est tout. Mais une chose est sure, l'enfant doit faire son dépistage. » Marie Laure, VIH-négatif, concubinage.

#### 3.5.3. … Et d'autres mères opposées au dépistage de l'enfant utilisent des stratégies

A l'opposé des premières, ces autres mères refusent le dépistage de leur enfant qu'elles partagent ou non le principe. Si parmi elles, certaines ont confié ne rien dire au conjoint, d'autres par contre ont affirmé requérir leur avis.

Les mères de cette dernière catégorie incriminent le message véhiculé lors du counseling pour justifier leur double refus d'informer le père et de dépister l'enfant. Elles font savoir que la sensibilisation sur le dépistage précoce les incrimine. Selon elles, le message les rendes seules ‘responsables’ de l'infection chez le nourrisson. Si l’époux est informé du test et que l'enfant est dépisté infecté, le père accusera la mère parce que préalablement informé, sensibilisé sur la TME qui révèle leur « responsabilité entière dans l'infection chez l'enfant ». Cela pourrait avoir des conséquences pour le couple mère-enfant. Par conséquent, elles préfèrent ne pas informer l’époux:

« … Avant je ne savais pas qu'on fait test de sida des enfants aussi. C'est aujourd'hui que j'ai appris qu'on peut faire [ … ]. Mais j'ai refusé parce que d'après ce que la femme a dit, si l'enfant a sida c'est comme si c'est sa maman qui lui a donné ça. Moi j'ai compris que si on fait le test et mon enfant a sida, c'est d'abord moi qui l'a contaminé. Donc, moi je ne peux pas aller informer son papa parce que je ne sais pas ce qui va se passer entre lui et moi si il accepte de faire le test de l'enfant et puis l'enfant a sida. ». BR 205, VIH-inconnu.

Bien qu'opposées au dépistage de leurs enfants, d'autres mères désirent en discuter avec leurs conjoints. Il faut informer l’époux par principe parce qu'il est le père/le chef de la famille. Cependant, pour cette catégorie de mères, si celui-ci est d'accord: elles refuseront sans le lui faire savoir. Ce qui est tout à fait possible parce que l’étude exigeait l'avis favorable des deux parents (Leroy *et al.* 2009) et les mères sont seules à se rendre aux consultations. Alors, fort de ces faits, elles pourront dire à leur époux que le test a été fait et que l'enfant n'est pas infecté.

Les mères usent donc de subterfuges pour ne point dépister leur enfant. La réaction des mères fait référence à l'expérience des échanges antérieurs sur le VIH/sida dans le couple. La vie conjugale est en effet un processus, elle est une construction et tient compte de l'intégration, de la compréhension mutuelle et de la considération des attitudes et comportements des conjoints. Selon J-C Kaufmann, ‘l'intégration conjugale est devenue lente et beaucoup plus complexe’ parce que le couple requiert consensus et par conséquent une compréhension mutuelle et une considération permanente du point de vue de l'autre. Le couple, marqué par une vie conjugale, n'est donc pas une réalité directement observable mais un processus, une construction par ses membres.

En conséquence, la construction du couple ne peut être achevée sans tenir compte des échanges sur toutes les questions ayant trait au couple et notamment la prévention du VIH. En ce qui nous concerne, cette construction devra prendre en considération la possibilité pour les conjoints d’échanger sur le dépistage pédiatrique. Ces échanges mettent en exergue les points de vue des conjoints, les laissent se découvrir et constituent pour eux une expérience qui servira à la décision (Dubet 1994; Benoïst 2002):

« … Je vais vous dire que mon mari m'a déjà montré qu'il est opposé au test. Et voici qu'ici à l'hôpital on vient de me dire que c'est important qu'on fasse le dépistage chez les enfants comme le mien. Cette information est nouvelle pour moi et aussi pour lui qui refuse déjà le test des adultes. Donc je vais [ … ] lui porter cette information [ … ] et lui va décider du reste. Mais si mon mari me dit, non je ne ferai pas dépister mon enfant tout simplement » Elisabeth, VIH-inconnu, refus test enfant.

## 4. Discussion: De l’émergence de nouvelles questions … 

### 4.1. Meres, principales mises en cause

Face au dépistage précoce pédiatrique, les mères sont interpellées car a priori responsables de l'infection chez l'enfant. Alors, pour certaines, il convient de dépister l'enfant. Par contre, pour les autres, il n'est pas question de dépister l'enfant. Ces différentes positions tiennent compte de l'expérience des couples face au dépistage VIH. Cette expérience est personnelle, dans son vécu comme ses implications et oriente la décision des mères d'associer ou non le père/conjoint pour le dépistage du nouveau-né.

Sans consulter le père, les mères (quelle que soit leur réaction face à la proposition du dépistage pédiatrique) savent les risques encourus. Néanmoins, celles qui refusent le test de l'enfant s'appuient sur le principe du dépistage pédiatrique (accord des *deux* parents). A l'opposé, l'accord requis des deux parents ou du père avant tout test place dans un dilemme les mères qui souhaitent faire dépister l'enfant: faut-il informer le père sachant que celui-ci va refuser ? Dans ce cas que faire, sachant que l'infection chez l'enfant conduira à une PEC et que le refus du test n'informe pas sur le statut VIH ?

En acceptant de faire le test de l'enfant à l'insu du père, la mère encourt des risques et en fait courir également à l'enfant en cas d'infection: (i) prise de TAR en catimini et probabilité grande d'inobservance chez l'enfant parce qu'impossibilité pour la mère de faire prendre toujours en cachette le TAR à l'enfant (ii) risque de morbidité et décès de l'enfant. La mère quant à elle court le risque d’être expulsée, rejetée/abandonnée avec l'enfant si le père venait à tout découvrir.

Face donc au dépistage pédiatrique, des constats sont à relever: de même que l'acceptation du test pédiatrique pour certaines mères conduit à la prise d'initiatives, le refus de faire dépister l'enfant pour les autres mères conduit aussi à prendre d'autres initiatives. Celles-ci prennent encrage dans l'expérience des unes et des autres, et de l'urgence du test pédiatrique. Dans l'un comme dans l'autre cas, se pose avec insistance la question du père.

### 4.2. conjoint, père obstacle et/ou facilitateur

Certaines mères pour des raisons déjà énumérées préfèrent contourner le père pour réaliser ou non le test pédiatrique. Toutefois, il n'en demeure pas moins qu'elles ont conscience de toute la place de l’époux/du père qui est un personnage central. Celui-ci peut parfois être considéré comme un frein quand il est susceptible de faire obstacle au dépistage et aussi moteur de la réalisation du test chez l'enfant, notamment dans le cas où la mère n'est pas consentante.

En conséquence, la question qui se pose est de savoir si le test pédiatrique doit continuer à être subordonné à la seule décision du détenteur de la puissance parentale, le père ? Face à son refus, doit-on avoir recours à un appui institutionnel donnant le pouvoir à la mère de décider?Si non, comment atteindre l'objectif de faire dépister précocement les enfants ? Comment impliquer le père dans ce processus sans nuire à la mère ? Quelles actions faut-il entreprendre pour une plus grande adhésion des parents au test ?

Autant de questions que suscite le sujet sur l'acceptabilité du dépistage précoce chez les enfants. Questions logiques qui méritent attention parce que ces enfants dépendent de leurs parents qui eux-mêmes posent des difficultés pour leur propre dépistage (Dédy & Tapé 1995; Oga 2006).

### 4.3. Reconsidération de l'infection

Les faits rendus par les enquêtés, objet des paragraphes suivants, peuvent être jugés de données subjectives parce qu'elles n'ont fait suite à aucune analyse sur-interprétative éloignant parfois de la pensée originelle de l'enquêté à ne pas perdre de vue. Ces faits prennent en considération le souhait des mères d’élaborer un schéma impliquant davantage le père, et situent sur la volonté des mères de faire des propositions facilitant le test précoce pédiatrique.

Ces propositions ont trait au statut particulier accordé au VIH/sida et aux informations disponibles dans le carnet de santé de la mère et de l'enfant. S'agissant de la première, il faut reconsidérer l'infection à VIH/sida comme une pathologie dont l'approche entre soignant-soigné ne devrait pas différer de l'approche soignant-patient dans le cas des autres maladies. La seconde proposition vise l'amélioration des informations disponibles dans le carnet de santé.

### 4.4. Amélioration des mentions du carnet de santé de la mère et de l'enfant

Cette amélioration est relative aux mentions à inscrire dans le carnet de santé et qui renseignent sur le dépistage de la mère et du nourrisson. Par là, les mères souhaitent aider à améliorer l'adhésion au test pédiatrique.

Faire mention de la réalisation du test VIH, de la mère et de l'enfant, dans le carnet de santé permet à l'avance aux conjoints d’échanger sur l'opportunité de le faire. De cette manière, avant même de se rendre dans une structure sanitaire pour une consultation, la mère sait quelle décision convient pour elle et l'enfant s'agissant du test de dépistage. Cette proposition des mères pose la question du choix de la stratégie nationale de proposition du diagnostic de l'infection.

En dehors du dépistage volontaire, la proposition de dépistage peut être faite selon deux stratégies. L'une d'entre elle est dite stratégie ‘Opt-out’ (dépistage implicite ou dire NON). Avec elle, l'acceptation du test est tacite, il revient au patient de manifester son refus s'il ne souhaite pas être dépisté. L'autre stratégie, ‘Opt-in’ (dépistage explicite ou dire OUI) a cours en Côte d'Ivoire. La proposition est faite sur place et exige un consentement. L'option ‘Opt-out’ est donc celle à laquelle semble adhérer les mères.

## 5. Conclusion

En définitive, serait-on en droit de faire le test chez les nourrissons quel que soit l'avis du père/de la mère ? Peut-il se faire sans l'avis du père ? Le dépistage pédiatrique devra-t-il exiger l'accord des deux parents ? Alors, quelles actions entreprendre pour amener les populations à y adhérer ?

La Côte d'Ivoire (Tenoh 2009) connait des mutations sociales et politiques relevant de la question du genre. Celles-ci, marquées par des changements dans les rapports homme/femmes (Marie 1997; Schatz 2005), conduisent à redéfinir les rôles et statuts dans le couple (Schatz 2005; Tenoh 2009). Cependant, ces changements peuvent-ils conduire à la couverture par le dépistage VIH des nourrissons ? Ainsi, le rôle de la mère doit être réinvesti et le père davantage intégré dans tous les aspects de la santé de l'enfant pour améliorer l'acceptation du dépistage pédiatrique. Toutes ces questions doivent être considérées pour développer une politique de dépistage précoce efficiente en Côte d'Ivoire.

La lutte contre l'infection par le VIH/sida est complexe et délicate par ses questions d'ordres social, économique, politique, éthique, culturel et idéologique (Benoïst 2002). Elle requiert, pour son contrôle, une anticipation dans « l'organisation de la lutte [ … ] et la prévision des réponses adaptées aux situations » (Desclaux & Raynaut 1997). Dans ce contexte, le dépistage précoce chez les nourrissons demeure une urgence pour une PEC rapide des cas d'infections. À ce niveau, deux constats sont à formuler. Le premier tient compte de l'avertissement de JA. Soumahoro pour qui de « nombreux obstacles rendent peu réaliste l'ensemble de la chaîne qui va de l'information au dépistage et à la PEC » (Desclaux & Raynaut 1997). Le second constat révèle selon certains auteurs que les actions dirigées vers les populations modifient leur perception du VIH/sida (Desclaux & Raynaut 1997). Et, pour les autres, les résultats obtenus guident des actions correctives (Hollos & Larsen 2004).

Dans une telle situation, une politique de proposition systématique du test aux nourrissons doit s'entourer de certaines garanties pour son succès. Il convient de procéder ainsi car la proposition du test est, le plus souvent, faite à la mère qui, même pour son propre test, s'affranchit difficilement de l'avis du conjoint (Oga 2006).

## Notes

1. On estime à environ 67% le nombre de personnes vivant avec le VIH en Afrique subsaharienne sur l'ensemble des PVVIH au monde (ONUSIDA, 2009). Ce chiffre a donc été obtenu par calcul associant le nombre total d'enfant infectés dans le monde (2,1 millions) et les 67%.

2. Cas es projets Pearl 2008, MTCT-Plus 2005, Ditrame-Plus 2001, Ditrame 1998.

3. Ces chiffres ont été obtenus par un calcul appliquant le taux d'acceptation du dépistage, la proportion de femmes enceintes infectées et le taux de transmission sans intervention de 25%.

4. Ce qui est contraire à la Recommandation OMS 2012, TasP, Treatment as Prevent, visant à prévenir la propagation de l'infection par le traitement des personnes dépistées infectées par le VIH.

5. Recommandation OMS, 2008: mise sous traitement ARV de tout enfant dépisté infecté par le VIH.

6. Le projet Pédi-Test ANRS 12165 a été initié en Côte d'Ivoire notamment à Abidjan (Hôpital général de Port Bouët, Hôpital général de Koumassi et Formation sanitaire Urbaine à base Communautaire d'Abobo Avocatier) et à Bonoua (Hôpital général) de juin 2008 à décembre 2009.

7. Est dit parent, tout géniteur des enfants de 6 à 26 semaines à qui la proposition du test pédiatrique précoce VIH a été faite. Le parent ou les parents représentent une seule personne parlant au nom de l'enfant, soit le père, soit la mère et non les deux. Le couple parent représente les deux géniteurs ensemble reçus lors de la proposition du test précoce VIH de leur enfant.

## References

[CIT0001] Baxerres C., Le Hesran J.-Y. (2004). Recours aux soins et fièvre chez l'enfant en pays Sereer ay Sénégal: entre contrainte économique et perception des maladies. Sciences Sociales et Santé.

[CIT0002] Benoïst J.

[CIT0003] Blanchet A., et Gotmann A. (1992).

[CIT0004] Coulibaly T. D., Msellati P., Vidal L. (2003). Essai clinique Ditrame (ANRS 049) visant à réduire la transmission mère-enfant du VIH à Abidjan. Presse Médicale.

[CIT0005] Dédy S., Tapé G. (1995).

[CIT0006] Desclaux A. (2004).

[CIT0007] Desclaux A., Raynaut C.

[CIT0008] Dubet F. (1994). Sociologie de l'expérience.

[CIT0009] Hassoum J. (2006).

[CIT0010] Hollos M., Larsen U. (2004). Which African Men Promote Smaller Families and Why? Marital Relations and Fertility in a Pare Community in Northern Tanzania. Social Science and Medicine.

[CIT0011] Kankasa C., Carter R. J., Briggs N. (2009). Routine Offering of HIV Testing to Hospitalized Paediatric Patients at University Teaching Hospital, Lusaka, Zambia: Acceptability and Feasibility. Journal of Acquired Immunodeficiency Syndrome.

[CIT0012] Leroy V., Brou H., Oga A. C. M. (2009).

[CIT0013] Marie A. (1997).

[CIT0014] Medley A., Garcia-Moreno C., Mc-Gill S. (2004). Rates, Barriers and Outcomes of HIV Serostatus Disclosure Among Women in Developing Countries: Implications for Prevention of Mother-to-Child Transmission Programmes. Bulletin of the World Health Organisation.

[CIT0015] Newell M. L., Coovadia H., Cortina B. M. (2004). Child Mortality and HIV Infection in Africa. A Review. AIDS.

[CIT0016] Oga A. C. M. (2006).

[CIT0017] Raynaut C., Muhongayire F. (1994). Connaître ou ignorer, dire ou taire: les ambiguïtés de l'annonce du statut sérologique chez les femmes de Kigali (Rwanda. Personnes atteintes: des recherches sur leur vie quotidienne et sociale.

[CIT0018] Rollins N., Mzolo S., Moodley T. (2009). Universal HIV Testing of Infants at Immunization Clinics: An Acceptable and Feasible Approach for Early Infant Diagnosis High HIV Prevalence Settings. AIDS.

[CIT0019] Schatz E. (2005). Take Your Mat and Go!: Rural Malawian Women's Strategies in the HIV/AIDS Era. Culture, Health and Sexuality.

[CIT0020] Suremain C.-É.D., Lefèvre P., Pécho I. (2000). Les relations du genre soumises à l’épreuve de la maladie: exemples boliviens et péruviens. Recherches Feminists.

[CIT0021] Tenoh A. (2009).

[CIT0022] Théry I. (1999). Une femme comme les autres: Séropositivité, sexualité et féminité. Séropositivité, vie sexuelle et risque de transmission du VIH.

[CIT0023] Tijou-Traoré A. (2009). Rôles des conjoints et pères à l’égard de la prévention du VIH à Abidjan (Côte d'Ivoire. Autrepart.

[CIT0024] Tijou-Traoré A., Groupe Ditrame Plus 3 Dialogue (2006). Gestion des risques de transmission du VIH et choix reproductifs au sein de couples sérodifférents résidant à Abidjan (Côte-d'Ivoire. Sexualité et procréation confrontées au sida dans les pays du Sud.

[CIT0025] Vidal L. (2000). Femmes en temps de sida.

